# rdml: A Mathematica package for parsing and importing Real-Time qPCR data

**DOI:** 10.1186/s13104-017-2533-9

**Published:** 2017-06-12

**Authors:** Ramiro Magno, Isabel Duarte, Raquel P. Andrade, Isabel Palmeirim

**Affiliations:** 1 Theoretical Biology and Bioinformatics, Utrecht University, Padualaan 8, 3584 CH Utrecht, The Netherlands; 2 CBMR, Centre for Biomedical Research, University of Algarve, 8005-139 Faro, Portugal; 3 CMBI, Center for Molecular and Biomolecular Informatics-NCMLS, Radboud University, Geert Grooteplein 28, 6525 GA Nijmegen, The Netherlands; 4 Regenerative Medicine Program, Department of Biomedical Sciences and Medicine, University of Algarve, 8005-139 Faro, Portugal

**Keywords:** Mathematica, Wolfram, RDML, PCR, qPCR, DNA, RNA

## Abstract

**Objective:**

The purpose and objective of the research presented is to provide a package for easy importing of Real-Time PCR data markup language (RDML) data to Mathematica.

**Results:**

Real-Time qPCR is the most widely used experimental method for the accurate quantification of gene expression. To enable the straightforward archiving and sharing of qPCR data and its associated experimental information, an XML-based data standard was developed—the Real-Time PCR data markup language (RDML)—devised by the RDML consortium. Here, we present *rdml*, a package to parse and import RDML data into Mathematica, allowing the quick loading and extraction of relevant data, thus promoting the re-analysis, meta-analysis or experimental re-validation of gene expression data deposited in RDML format.

## Background

Real-Time quantitative polymerase chain reaction (qPCR) is a sensitive and powerful experimental method routinely used in molecular biology labs for quantifying DNA and RNA. qPCR instruments collect a large set of data per run, providing the basis for the quantification and validation of PCR amplification products. An instrument-independent format to store and exchange these data collections was published in 2009 as the first version of the Real-Time qPCR data markup language (RDML) [1]. This is an XML-based data standard, containing not only the measurements from the thermocycler, but also the metadata necessary to fully characterize the experimental conditions used to obtain those results. However, it is not trivial to automatically extract all the relevant information from such a comprehensive and technique-specific XML-based format.

## Main text

Accordingly, we have developed the rdml.m package, which extends Wolfram’s language functionality by allowing RDML version 1.2 files to be readily imported and loaded into Mathematica™ . The data from all mandatory and optional elements are validated first, and subsequently returned as a Dataset object; allowing the user to take full advantage of the recently introduced database-like operations for Dataset objects in Mathematica.

Making RDML data easily accessible is an important step toward promoting the peer-validation of published datasets managed by the RDML consortium (http://www.rdml.org/). There are a few software applications compatible with RDML, namely the *RDML Ninja* [2], *qbase+* [3], *RDML R package* (https://github.com/kablag/RDML/), and *LinRegPCR* [4], of which only the first and the last fully support RDML version 1.2. Our package brings these data to Mathematica users wishing to take advantage of the symbolic character and new data query potential of the Wolfram Language, hence broadening the spectrum of software able to access, analyse and manipulate RDML data.

Two of the main advantages of this *rdml* package are: (1) the fact that it does not require the user to become familiarized with the sizeable RDML schema, and (2) it does not require an extensive learning of new package-specific functions. Instead, we have expanded the built-in Import function from Mathematica, making it directly capable of importing RDML files. For this, we registered specific import functions for the various RDML master elements: dateMade, dateUpdated, id, experimenter, documentation, dye, sample, target, thermalCyclingConditions and experiment. This registration is achieved by using the function ImportExport‘RegisterImport as indicated in (https://reference.wolfram.com/language/tutorial/DevelopingAnImportConverter.html). Additionally, we overload Import to allow it to automatically recognize the file extensions .rdml and .rdm.

The returned Dataset expression is a nested structure of lists and associations. The hierarchy in the returned object preserves the original hierarchy defined in the RDML schema v1.2. Each XML element that is not a leaf element, is either represented by an Association or a List. We use Associations for lists of RDML elements that have an id XML-attribute, using the id value as Key; XML elements with no id are represented as Lists. Leaf elements are converted to either Symbol (True or False), Integer, Real, String or DateObject. Special numeric codes such as INF are appropriately dealt with, being converted to Mathematica equivalent expressions, i.e., Infinity. Missing or non-schema-compliant elements are converted to Missing[NotAvailable].

### RDML validation

Another important feature of this package is the validation of the input file against the RDML schema version 1.2. This step is optional by setting ValidateAgainstXSD to True when importing. For this, we leverage on Java’s support for XML schema by using J/Link in Wolfram (http://reference.wolfram.com/language/JLink/tutorial/CallingJavaFromTheWolframLanguage.html). Additionally, *rdml* is partially compatible with versions 1.0 and 1.1, only to the extent of the overlap between the schemas.

### Package overview

Once loaded, the *rdml* package extends the Import function from Mathematica, enabling it to read and parse RDML files. The importer returns a Dataset object containing all valid data, which is then easily browsable using Mathematica’s newly implemented query language for Datasets.

### Loading the rdml.m package

First, the package file must be made available to Mathematica. For this, make sure that the current working directory is where the rdml.m file is located, and simply load the package with: Get [“rdml.m”]; once loaded, the user can access the documentation notebook via the function: HelpPageRDML[].

### Importing RDML data

To import an RDML file use the built-in Mathematica Import function. For example, to import one of the sample dataset files included with the package, the rpa.rdml file (which can also be found in the datasets folder in https://github.com/ramiromagno/rdml), simply run: Import[“datasets/rpa.rdml”].

**Fig. 1:**
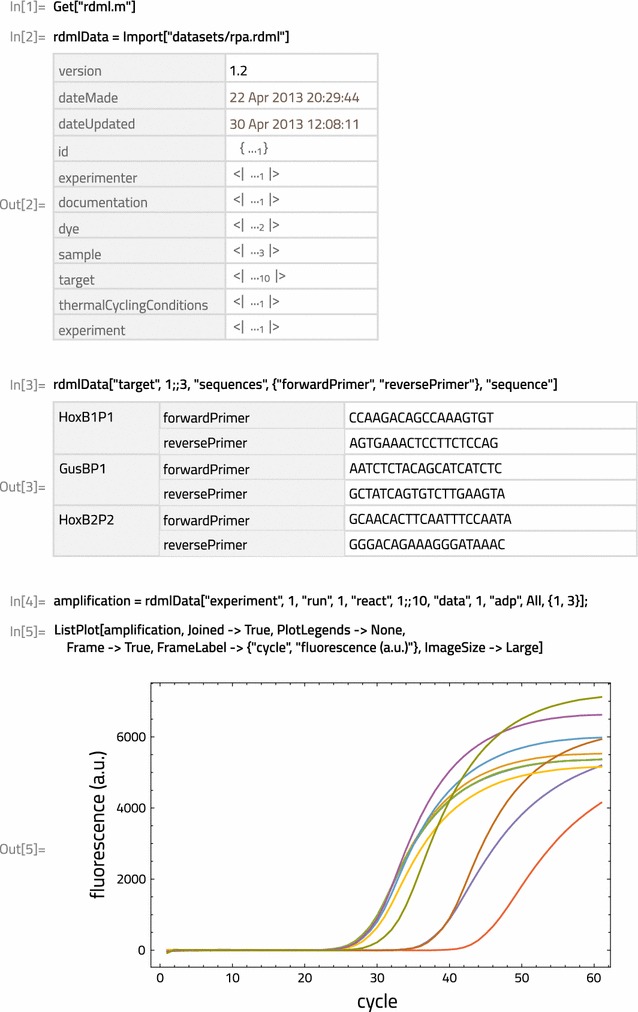
Screenshot of a typical rdml usage session in Mathematica

Next, the user can browse and freely explore the data within Mathematica. In Fig. 1, Out[2] shows the returned summarized Dataset object for the rpa.rdml. The main RDML elements are all present, as well as the schema version of the input file. Values for most elements are long expressions, hence shown in abbreviated form. Short expression values, such as those for dateMade and dateUpdated, can be fully displayed and are automatically returned as DateObjects. Once returned, the Dataset object structure can be easily traversed by using Dataset queries or Part operations, as illustrated in In[3]. This example shows how to access the nucleotide sequences of the primers used to amplify three of the PCR targets.

### Discussion

The *rdml* importer is an open source Wolfram package that validates and automatically loads RDML data into Mathematica. Standard RDML files are generated by most of the widely used qPCR equipments. This package makes archived qPCR data, and all of its experimental settings, readily available to Mathematica users without the additional burden of becoming familiarized with the RDML schema and having to learn new package-specific functions.

## Limitations

Although the package here presented is free and open source software, Mathematica itself is not open source software.

## Abbreviations

RDML: Real-Time PCR data markup language
qPCR: Real-Time quantitative PCR
PCR: polymerase chain reaction
XML: extensible markup language
